# *Cis*-acting mutation affecting *GJA5* transcription is underlying the *Melanotic* within-feather pigmentation pattern in chickens

**DOI:** 10.1073/pnas.2109363118

**Published:** 2021-10-04

**Authors:** Jingyi Li, Mi-Ok Lee, Junfeng Chen, Brian W. Davis, Benjamin J. Dorshorst, Paul B. Siegel, Masafumi Inaba, Ting-Xin Jiang, Cheng-Ming Chuong, Leif Andersson

**Affiliations:** ^a^Department of Veterinary Integrative Biosciences, College of Veterinary Medicine and Biomedical Sciences, Texas A&M University, College Station, TX 77843;; ^b^Key Laboratory of Agricultural Animal Genetics, Breeding, and Reproduction of Ministry of Education, College of Animal Science and Technology, Huazhong Agricultural University, 430070 Wuhan, China;; ^c^Department of Animal and Poultry Sciences, Virginia Polytechnic Institute and State University, Blacksburg, VA 24061;; ^d^Science for Life Laboratory, Department of Medical Biochemistry and Microbiology, Uppsala University, SE-751 23 Uppsala, Sweden;; ^e^Department of Pathology, University of Southern California, Los Angeles, CA 90033;; ^f^Department of Animal Breeding and Genetics, Swedish University of Agricultural Sciences, SE-750 07 Uppsala, Sweden

**Keywords:** melanotic, patterning, GJA5, gap junction protein, IBD mapping

## Abstract

*Melanotic* (*Ml*) is a mutation in chickens that extends black (eumelanin) pigmentation in normally brown or red (pheomelanin) areas, thus affecting multiple within-feather patterns [J. W. Moore, J. R. Smyth Jr, *J. Hered.* 62, 215–219 (1971)]. In the present study, linkage mapping using a back-cross between Dark Cornish (*Ml*/*Ml*) and Partridge Plymouth Rock (*ml*^*+*^*/ml*^*+*^) chickens assigned *Ml* to an 820-kb region on chromosome 1. Identity-by-descent mapping, via whole-genome sequencing and diagnostic tests using a diverse set of chickens, refined the localization to the genomic region harboring *GJA5* encoding gap-junction protein 5 (alias connexin 40) previously associated with pigmentation patterns in zebrafish. An insertion/deletion polymorphism located in the vicinity of the *GJA5* promoter region was identified as the candidate causal mutation. Four different *GJA5* transcripts were found to be expressed in feather follicles and at least two showed differential expression between genotypes. The results showed that *Melanotic* constitutes a *cis*-acting regulatory mutation affecting *GJA5* expression. A recent study established the *melanocortin-1 receptor* (*MC1R)* locus and the interaction between the MC1R receptor and its antagonist agouti-signaling protein as the primary mechanism underlying variation in within-feather pigmentation patterns in chickens. The present study advances understanding the mechanisms underlying variation in plumage color in birds because it demonstrates that the activity of *connexin 40/GJA5* can modulate the periodic pigmentation patterns within individual feathers.

## Significance

The molecular mechanisms underlying pigmentation patterns in animals is to a large extent an unresolved mystery in biology. For example, compared with mammals, birds show a stunning diversity in pigmentation patterns. This study advances the knowledge concerning the mechanisms creating periodic pigmentation patterns in individual feathers. We show that a mutation upstream of *GJA5* encoding a gap-junction protein is causing the Melanotic phenotype in domestic chickens. *Melanotic* affects within-feather pigmentation patterns by enhancing the contrast between dark- and light-colored regions in the feather. The result implies that cell–cell communications between melanocytes and other cells in the feather follicle play a critical role for pattern formation.

Birds exhibit a remarkable diversity in plumage color, including the intensity and type of pigmentation (dark eumelanin versus red/yellow pheomelanin) as well as presence of carotenoid and porphyrin pigmentation, and structural colors (1). The diversity is further enhanced by patterning across body regions and by within-feather patterning. The domestic chicken is the primary animal model for genetic studies of pigmentation patterns due to extensive collection of mutations affecting plumage color in this species (1). Eight major types of within-feather patterns have been described in the domestic chicken: stippling (wild-type), autosomal barring, pencilling, single and double lacing, spangling, mottling, and sex-linked barring (1, 2). Several genes affecting this phenotypic variation have been identified. *Extension/melanocortin-1 receptor* (*MC1R*) is the major feather-patterning gene in chickens and different alleles at this locus are required for all periodic feather patterns except sex-linked barring (1, 3). Furthermore, mottling is caused by a mutation in *EDNRB2* (4), sex-linked barring is caused by the combined effect of regulatory and coding changes in *CDKN2A* (5, 6), and a deletion upstream of *SOX10* is causing the *Dark Brown* allele associated with both autosomal barring and spangling (3, 7).

**Fig. 1.**
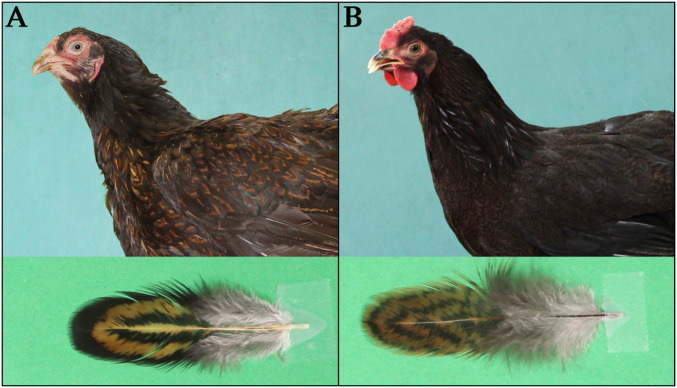
Within-feather patterns in chicken explored in this study. Pictures of Dark Cornish (*Ml/Ml*) hen (*A*) and Partridge Plymouth Rock (*ml*^*+*^*/ml*^*+*^) hen (*B*), and their individual feathers from breast, showing the double-lacing (*A*) and pencilling (*B*) patterns.

Several of the within-feather patterns in chickens however, are caused by the combined effect of variant *MC1R* alleles and mutations at other pigmentation loci (1). One of these is *Melanotic* (*Ml*), which enhances feather eumelanization and extends eumelanin into areas containing pheomelanin pigmentation in the wild-type (8). It contributes to the within-feather patterns single lacing (an outer ring of eumelanin that conforms to the edge of the feather), double lacing (two concentric eumelanic rings on a background pigmentation, that the outer ring conforms to the edge of the feather while the inner ring is separated from the outer by a ring of background pigmentation) (Fig. 1*A*), and spangling (a V-shaped eumelanic spangle located the distal end of the feather). *Melanotic* shows incomplete dominance over wild-type and it has been mapped to chicken chromosome 1 based on genetic linkage to the *Dark Brown/SOX10* locus (9).

The aim of the present study was to identify the gene for *Melanotic* using a back-cross between Dark Cornish (*Ml/Ml*), showing a typical double-lacing pattern expressed in females (Fig. 1*A*), and Partridge Plymouth Rock chickens (*ml*^*+*^*/ml*^*+*^) showing the pencilling pattern (Fig. 1*B*), combined with analysis of publicly available whole-genome sequencing (WGS) data. We demonstrate that *Melanotic* is caused by an insertion/deletion polymorphism located at the 5′end of the *gap junction protein α5* gene (*GJA5*) encoding connexin 40 and that *Melanotic* is a *cis*-acting regulatory mutation affecting *GJA5* expression.

## Results

### Linkage Mapping Assigns *Melanotic* to an 820-kb Region.

A linkage mapping population consisting of 2 Partridge Plymouth Rock males, 4 Dark Cornish females (*Ml/Ml*), 17 F_1_ females, and 126 female progenies was established by back-crossing the F_1_ females (*Ml/ml*^*+*^) to Partridge Plymouth Rock males (*ml*^*+*^*/ml*^*+*^). Phenotyping was carried out in female offspring only, because the pigmentation pattern is not visible in males. Clear pencilling or double-lacing phenotypes were observed in 60 and 43 back-cross females, respectively. The remaining 23 back-cross females were assigned to an intermediate group. The strategy for the identification of the *Melanotic* gene follows essentially Li et al. (10). Two back-cross DNA pools were constructed, Pool_pencilling and Pool_double_lacing; the back-cross individuals with an intermediate phenotype were not included in these pools. Two parental line DNA pools were also constructed (Pool_Cornish and Pool_Plymouth_Rock). The two back-cross pools were analyzed using a high-density 600K chicken SNP genotyping array, and all four pools were subjected to WGS in order to map and directly identify candidate mutations for *Melanotic*.

**Fig. 2.**
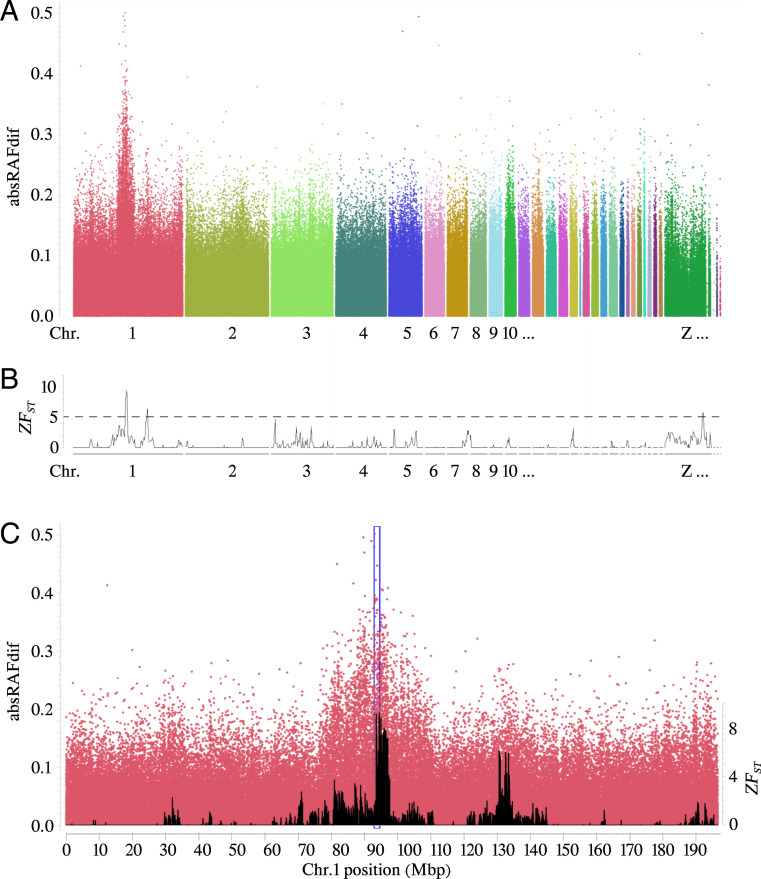
Linkage mapping assigns *Melanotic* to an 820-kb interval on chromosome 1. Genomic positions are given according to the GalGal6 assembly. (*A*) Genome-wide absRAFdif values based on the contrast between the pools of back-cross individuals showing pencilling or double-lacing feather patterns for all 600k SNPs, plotted against their genomic location. (*B*) Genome-wide *ZF_ST_* values for the same contrast but based on whole genome sequencing. (*C*) absRAFdif values for SNPs on chromosome 1 (red dots). The 1.6-Mb region harboring *Melanotic* according to the first round of linkage mapping is highlighted by a blue box. *ZF_ST_* values based on WGS are indicated in black.

The maximum possible absolute difference in relative allele frequencies (absRAFdif) between pools is 0.5, which occurs when the parental lines are fixed for different alleles. A single peak of high absRAFdif values was detected on chromosome 1 (Fig. 2*A*) when comparing the two back-cross pools based on the SNP-chip data. It overlaps the peak with highest *ZF*_*ST*_ values calculated using WGS data from the same groups (Fig. 2 *B* and *C*). By genotyping six selected SNPs (*SI Appendix*, Table S1) in all back-cross individuals with clear pencilling or double-lacing patterns, a first round of linkage mapping assigned *Ml* to a 1.6-Mb region defined by rs314066698 (93.0 Mb) and rs314825166 (94.6 Mb) (*SI Appendix*, Table S1, genome coordinates according to the GalGal6 assembly). Linkage mapping also revealed that 9 of 60 back-cross individuals with the pencilling pattern appeared as double recombinants between *Ml* and these two closely linked SNPs, which suggest that they should be *Ml*/*ml*^*+*^ instead of *ml*^*+*^/*ml*^*+*^. This result is consistent with the previous report that *Ml* shows incomplete dominance over wild-type (8). Only five individuals were single recombinants between *Ml* and either of these two SNPs. These five recombinant individuals were used for a second round of linkage mapping using 10 more SNPs identified by WGS. These SNPs were fixed for different alleles in the parental lines and narrowed down the candidate region to 820 kb defined by rs15347589 at 93,641,597 bp and rs317781986 at 94,458,113 bp (*SI Appendix*, Table S2).

### Identification of a 21.4 kb Identical-by-Descent Region Associated with *Melanotic*.

**Fig. 3.**
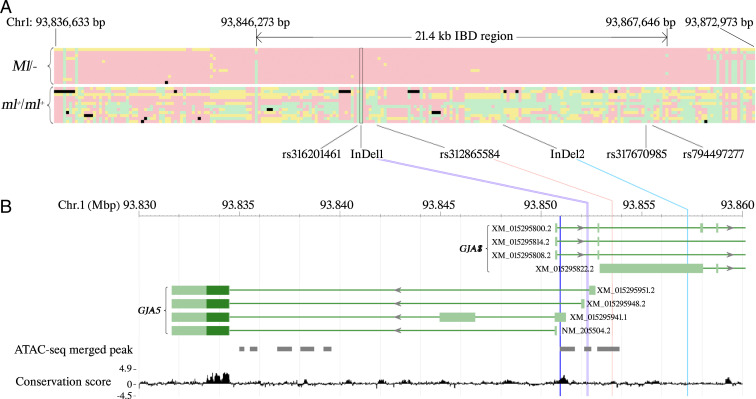
IBD mapping and identification of candidate mutations for *Melanotic* (*Ml*). Genomic positions refer to the GalGal6 assembly. (*A*) Haplotypes within and flanking the 21.4-kb IBD region identified by the WGS data of 12 samples from chickens carrying *Ml* (*Upper*) and 12 of 82 samples from chickens without *Ml* (*Lower*). Each cell represents the genotype of one polymorphism: green means homozygote of reference allele, red means homozygote of alternative allele, yellow means heterozygote, black means no call. Each row represents one sample. Each column represents one polymorphic site; the sites that are fixed for the reference allele in the top 12 samples are excluded. The six candidate mutations are indicated. (*B*) Localization of three candidate mutations, in relation to the *GJA8* and *GJA5* transcripts. Only the 5′ parts of the *GJA8* transcripts are shown. Dark green bars represent coding regions while light green bars represent UTRs. The dark blue vertical line represents a predicted transcription start site of *GJA5* (15). The gray solid boxes represent ATAC-seq merged peaks (11). The 77 vertebrates basewise PhyloP conservation scores (https://hgdownload.soe.ucsc.edu/goldenPath/galGal6/phastCons77way/) are shown at the bottom.

We searched for an identical-by-descent (IBD) region within the 820-kb region on chromosome 1 using WGS data from 10 samples of chickens showing the *Melanotic* pattern, including three samples with double-lacing pattern, two with silver spangled, and five with single lacing, together with two White Crested Black Polish individuals (*Ml*/− samples in *SI Appendix*, Table S3). This analysis was based on the assumption that chickens carrying the same causal mutations should share an IBD region in the vicinity of the mutations. As a result, a 21.4-kb IBD region (chr1:93,846,273 to 93,867,646) (Fig. 3*A*) was identified among these 12 samples. Within this region, only six sequence variants, including four SNPs and two InDels, were completely associated with the *Ml* haplotype, and none of these *Ml*-associated sequence variants was found to be homozygous in the 82 samples showing nonmelanotic patterns (*ml*^+^/*ml*^+^ samples in *SI Appendix*, Table S3, including pencilling, autosomal barring, and stippling).

### Identification of a Single Candidate Causal Variant Associated with *Melanotic*.

The six candidate mutations (*SI Appendix*, Table S4) were genotyped in a collection of 101 DNA samples classified as *Ml/−* or *ml*^+^/*ml*^+^ (*SI Appendix*, Table S5). Combining this result and all the WGS data used for IBD mapping, InDel1 was the only sequence variant that showed complete association with *Ml* and was absent in all *ml*^+^/*ml*^+^ samples (Table 1). The other five closely associated mutations were less likely to be causal, because the variant allele (associated with *Ml*) was also found on wild-type haplotypes (Table 1). However, it should be noted that SNPs rs312865584 and rs317670985 were excluded because single individuals were heterozygous for these SNPs. InDel1 and all the other closely associated sequence variants are located near the closely linked paralogs *GJA5* and *GJA8*, but none changed the coding sequence (Fig. 3*B*), suggesting that *Melanotic* is caused by regulatory mutations affecting the expression of one or both of these genes.

**Table 1.** Summary of genotypes of candidate mutations from WGS analysis and diagnostic tests

Breed*	Candidate mutations and their nucleotide positions (bp) on chromosome 1 based on GalGal6						*Ml* genotype^†^
	rs316201461	InDel1	rs312865584	InDel2	rs317670985	rs794497277	
	93,851,717	93,852,277	93,853,513	93,857,288	93,865,772	93,866,099	
White Crested Black Polish (*n* = 1)	Het	Mutant	Mutant	Mutant	Mutant	Mutant	*Ml*/−
All other *Melanotic* chickens (*n* = 58)	Mutant	Mutant	Mutant	Mutant	Mutant	Mutant	*Ml*/−
Red junglefowl (*n* = 1)	Het	WT	WT	WT	WT	Mutant	*ml*^+^/*ml*^+^
Red junglefowl (*n* = 2)	Het	WT	WT	WT	WT	WT	*ml*^+^/*ml*^+^
Red junglefowl (*n* = 1)	WT	WT	Het	WT	WT	Het	*ml*^+^/*ml*^+^
Partridge Cochin (*n* = 1)	Het	WT	WT	Het	Het	Het	*ml*^+^/*ml*^+^
Partridge Cochin (*n* = 1)	WT	WT	WT	Het	WT	WT	*ml*^+^/*ml*^+^
Nonmelanotic chickens (*n* = 6)	Het	WT	WT	WT	WT	WT	*ml*^+^/*ml*^+^
Nonmelanotic chickens (*n* = 11)	WT	WT	WT	WT	WT	Het	*ml*^+^/*ml*^+^
All other nonmelanotic chickens (*n* = 113)	WT	WT	WT	WT	WT	WT	*ml*^+^/*ml*^+^

* Breeds that do not show periodic feather patterns like Buff, Columbian, and Black were not included in this comparison because their *Ml* genotype is unknown.

^†^ Inferred from plumage phenotype.

In InDel1 a 41-bp sequence was replaced by a 38-bp sequence of unknown origin with no sequence homology in public databases (*SI Appendix*, Table S4). The ancestral 41-bp sequence has an average PhyloP conservation score of 0.48 (Fig. 3*B*) based on 77 vertebrates (https://hgdownload.soe.ucsc.edu/goldenPath/galGal6/phastCons77way/), which suggests that InDel1 may affect one or more conserved element. It is also within an ATAC-seq (assay for transpose-accessible chromatin with high-throughput sequencing) merged peak (11) detected in liver and T cell samples from White Leghorn chickens (Fig. 3*B*), supporting that InDel1 is located in a regulatory domain. The rs312865584 site also has a relatively high PhyloP conservation score (1.15) and is within another ATAC-seq merged peak. The InDel2 (6 bp) is not conserved (average conservation score is −0.26) and is not overlapping an ATAC-seq peak. TRANSFAC (12) analysis predicted that the variant InDel1 allele disrupts the binding sites of TCF-3 and TCF-7, while it creates new binding sites for GATA3, GATA4, LHX3, MEF-2D, and PMX1. Among them, *GATA3* is the only gene known to be expressed during feather development (13), and GATA4 is the only protein previously reported to interact with the proximal promoter of *GJA5* in rat (14).

The incomplete dominance of *Ml* was confirmed by genotyping InDel1 in the back-cross individuals. Ten of the 19 progenies scored as intermediate were genotyped as *Ml/ml*^*+*^ based on InDel1. Furthermore, we identified incomplete penetrance; nine back-cross individuals genotyped as *Ml/ml*^*+*^, both based on haplotype analyses and by genotyping InDel1, were phenotyped as nonmelanotic and appeared as double recombinants (see above) in the linkage analysis. Thus, we estimate a penetrance of about 85% for *Ml/ml*^*+*^ heterozygotes because 53 of 62 chickens showed a phenotypic effect of the *Ml* allele (43 double lacing and 10 intermediate).

### *Melanotic* Is Associated with Differential Expressions of Multiple *GJA5* Transcripts.

We explored the gene-expression patterns of *GJA5* and *GJA8* transcripts from seven different tissues, including skin and feather follicles from adult Dark Cornish (*Ml/Ml*) and Partridge Plymouth Rock (*ml*^*+*^*/ml*^*+*^) hens. We detected no expression of *GJA8* transcripts using four different primer pairs (*SI Appendix*, Table S6) in skin or in feather follicles. Therefore, it is unlikely that *GJA8* function is related to the Melanotic phenotype.

**Fig. 4.**
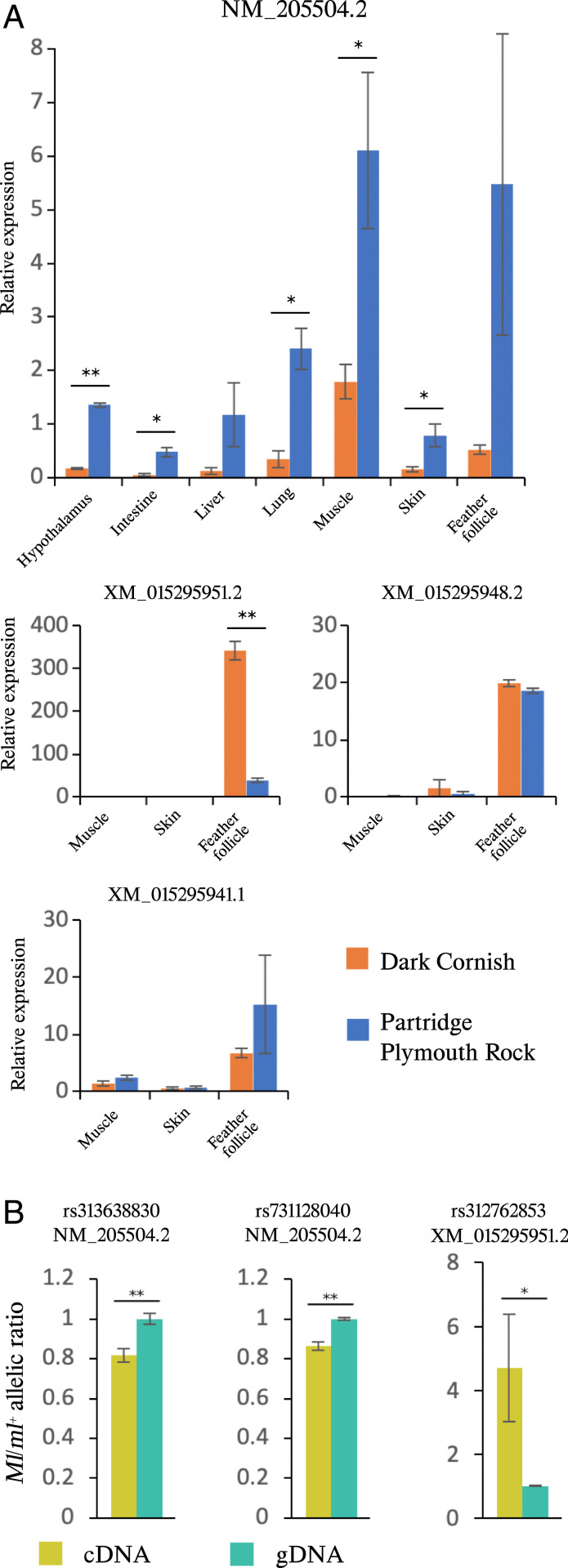
*Melanotic* is associated with differential expressions of multiple *GJA5* transcripts. Data are represented as mean ± SEM. (*A*) Results of qRT-PCR analysis of *GJA5* transcripts using tissues from adult Dark Cornish (*Ml/Ml*) and Partridge Plymouth Rock (*ml*^*+*^*/ml*^*+*^) hens. Feather follicle tissues were collected from developing feathers. For each transcript, the relative expression is reported as proportion to the expression level in the skin of one of the Partridge Plymouth Rock hens. Gene expression was normalized against *ACTB* expression; *n* = 2 for each data point. (*B*) *GJA5* expression was examined in feather follicles from *Ml*/*ml*^*+*^ heterozygotes. SNP markers and the associated transcripts are labeled on top of each figure. Peak height of each allele was quantified using the PeakPicker 2 software (34). The *y* axis shows the ratio of the peak height of the *Ml* allele over the *ml*^*+*^ allele. The three cDNA and three genomic DNA (gDNA) samples came from the same individuals (*n* = 3). *P < 0.05; **P < 0.01.

Four *GJA5* transcripts that only differ with regard to which untranslated exon 1 is used (i.e., they encode the same protein sequence) have been annotated in chickens (Fig. 3*B*) and we examined their expression (Fig. 4*A*). The *GJA5* transcript NM_205504.2 was detected in each of the seven tissues with consistently higher expression in Partridge Plymouth Rock (*ml*^*+*^*/ml*^*+*^) hens than in Dark Cornish (*Ml/Ml*) chickens (Fig. 4*A*). For the remaining three *GJA5* transcripts, we focused on the expression in skin and feather follicles because they are most relevant for the Melanotic phenotype; muscle tissue was used as control. Expression of all three transcripts were primarily detected in feather follicles (Fig. 4*A*). The expression of the XM_015295951.2 transcript was about ninefold higher in Dark Cornish *Ml/Ml* homozygotes than in wild-type Partridge Plymouth Rock (*ml*^*+*^*/ml*^*+*^) hens (Fig. 4*A*) (*P* < 0.01). Moreover, also transcript NM_205504.2 exhibited significant differential expression, but with an opposite trend (Fig. 4*A*). Based on the raw qPCR data, NM_205504.2 should be the dominant transcript because its average Ct value in feather follicle samples of Partridge Plymouth Rock was 22.9, while the corresponding value for XM_015295951.2 was 28.9. This difference implies that the level of expression for NM_205504.2 is more than 60-fold higher than XM_015295951.2 in wild-type feather follicle samples.

### *GJA5* Expression Shows Allelic Imbalance.

We crossed a red junglefowl male (*ml*^*+*^*/ml*^*+*^) with a Silver Sebright hen (*Ml/Ml*) to generate heterozygous offspring and investigate the relative expression of the two alleles within individuals. Sanger sequencing confirmed that the F_1_ individuals were heterozygous *Ml/ml*^*+*^, and heterozygous for three linked markers present in *GJA5* transcripts: rs313638830, rs731128040, and rs312762853. Therefore, these markers were used to test for the presence of allelic imbalance of *GJA5* expression. The results, based on the feather follicles, revealed a small but statistically significant down-regulation of NM_205504.2 from the *Ml* allele compared to the wild-type allele (Fig. 4*B*). In contrast, the XM_015295951.2 transcript showed almost a fivefold higher expression from the *Ml* than the wild-type *ml*^*+*^ allele (Fig. 4*B*). Thus, the results are consistent with the difference in expression patterns for these two transcripts based on qRT-PCR analysis of mRNA from pure line chickens (Fig. 4*A*). Taken together, the results provide strong evidence that *Melanotic* constitutes a *cis*-acting regulatory variant affecting *GJA5* expression.

### Reporter Assays Support an Effect on Transcriptional Regulation.

The predicted transcription start site of chicken *GJA5* (15) is 1.3 kb downstream of InDel1, 2.6 kb downstream of SNP rs312865584, and 6.3 kb downstream of InDel2 (Fig. 3*B*). We generated reporter constructs mimicking the *Ml* and wild-type *ml*^*+*^ haplotypes as regards the InDel1 and rs316201461 polymorphisms. The transfection experiments using DF40 fibroblast cells and these constructs resulted in a small (about 20%), but significant elevated expression from the *Ml* haplotype (*SI Appendix*, Fig. S1).

### *GJA5* Is Expressed in the Collar and Ramogenic Zone of Feather Follicles, in Both Melanoblasts and Keratinocytes.

**Fig. 5.**
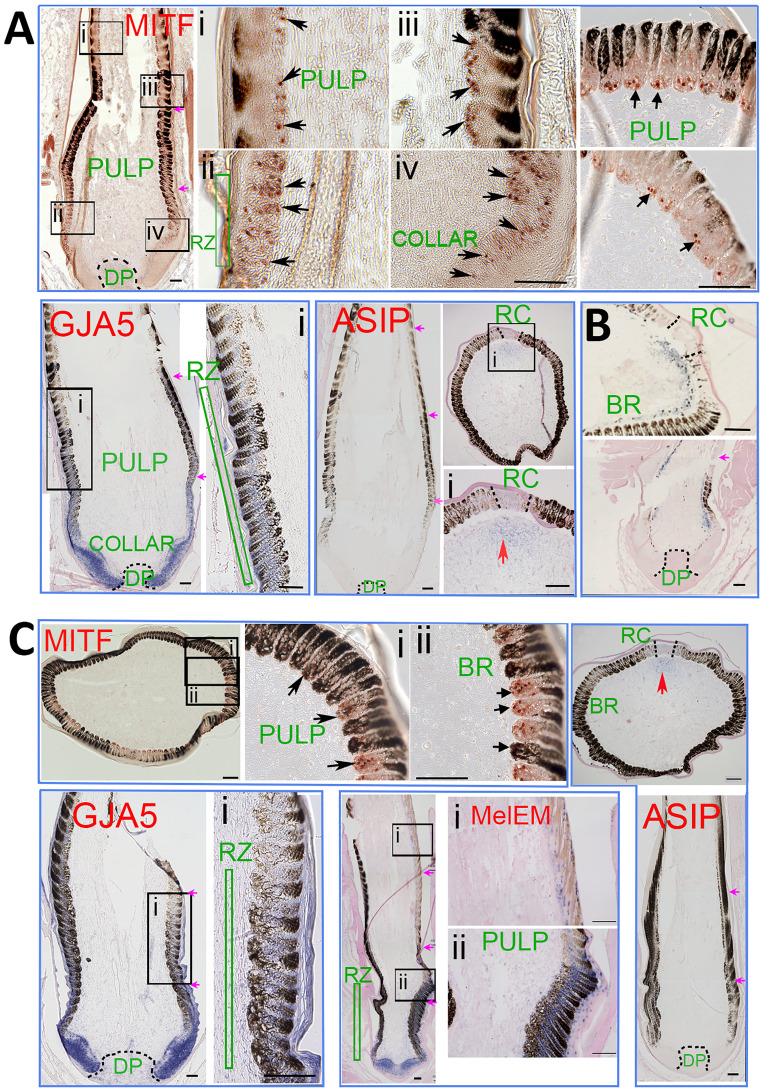
Molecular expression during feather formation. Immunostaining and ISH of Partridge Plymouth Rock feather follicles (*A*), Fayoumi feather follicles (*B*), and Dark Cornish feather follicles (*C*). MITF is based on immunostaining. GJA5, melEM, and ASIP are based on ISH. (*A*) MITF^+^ cells (red nucleus staining) are present in the basal layer of the feather filament epidermis in longitudinal feather sections (arrows in *A*, Mitf-i, ii, iii, and iv) and cross-section (*Right*) of both eumelanin and pheomelanin regions. GJA5 is expressed (blue color) in keratinocytes in collar and ramogenic zones. GJA5 is also expressed in melanocytes in the ramogenic zone in both the eumelanin and pheomelanin zones, but with decreased expression in the more differentiated barb ridges. ASIP is absent in the pulp in longitudinal sections. Cross-sections show that ASIP is weakly expressed in the peripheral pulp adjacent to the rachis region. (*B*) Fayoumi chicken feathers with autosomal barring pattern (3) are shown for comparison. Both cross and longitudinal sections show lower ASIP expression in the peripheral pulp adjacent to the eumelanin region. (*C*) MITF immunostaining (*Upper*) shows positive melanoblasts in both eumelanin and pheomelanin regions in Dark Cornish feather follicles. GJA5 and ASIP expression patterns are similar to the expression patterns shown in Partridge Plymouth Rock feather follicles (*A*). MelEM (blue nucleus staining) is expressed in melanoblasts in the distal collar and ramogenic zone, with strong expression in the eumelanin region and weak expression in the pheomelanin region. For feather follicle components, please refer to Fig. 6*A*. BR, barb ridge; DP, dermal papilla; RC, rachis; RZ, ramogenic zone. (*A*) MITF, GJA5, ASIP panels; (*B*) *Lower* panel; and (*C*) GJA5, MelEM *Left* panel, and ASIP panels are photomontages in which spliced junctions are indicated by purple arrows. (Scale bars in all panels, 100 μM.)

**Fig. 6.**
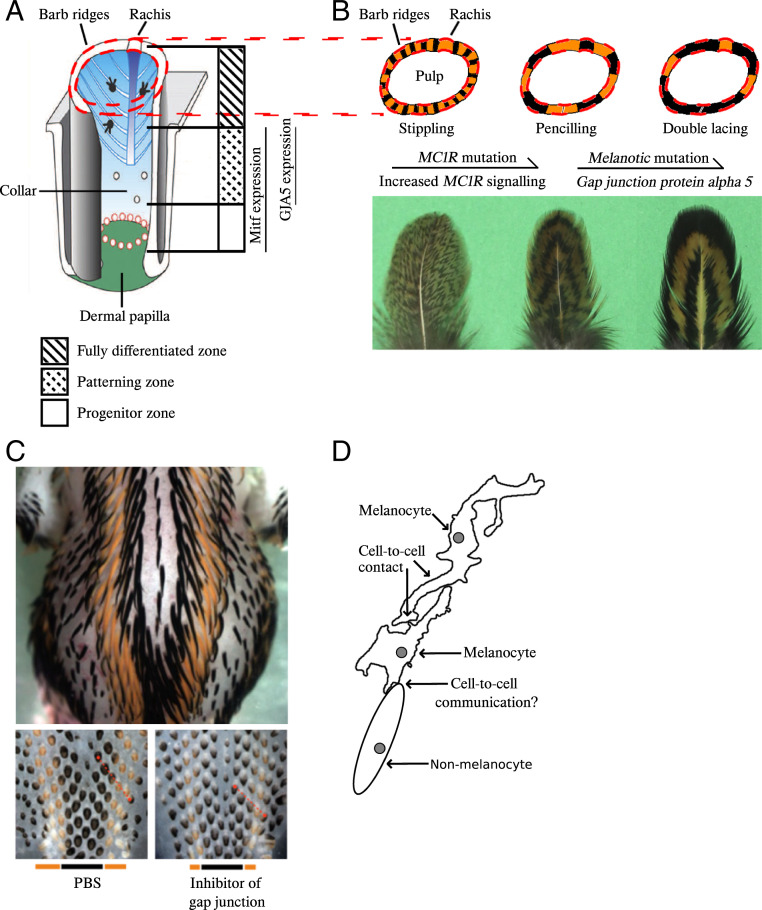
Gap junctions may play key roles in both within-feather and across-body pigment pattern formation. (*A*) Schematic drawing showing growing feather follicle structures [modified from Lin et al. (17)], and the corresponding regions where MITF and GJA5 are expressed based on this study. (*B*) Cross-section of growing feather follicles (*Upper*, schematic drawing) and the grown-up feathers (*Lower*, photos) for comparison between the three within-feather patterns and how mutations in *MC1R* and *GJA5* act additively to enhance expression of eumelanin. (*C*) Photos of Japanese Quail embryos [from Inaba et al. (19)]. Dorsal view of an E10 embryo shows across-body pattern (*Upper*), which was affected by gap-junction activity (*Lower*, E7). The bottom bars show the width of the eumelanin and pheomelanin stripes. The top panel photograph was taken at 1x magnification while the two bottom panels were taken at 3× magnification on a Nikon SMZ 1500 microscope. (*D*) Schematic drawing of possible ways for how gap junction proteins may be involved in cell-to-cell communication and thus affect pattern formations [modified from Inaba et al. (19)].

We examined the expression of microphthalmia-associated transcription factor (MITF), agouti-signaling protein (ASIP), and GJA5 in feather follicles during the formation of the periodic pigmentation stripes in pencilling and double-lacing feathers (Fig. 5). Both keratinocyte and melanocyte stem cells reside in the follicle base (16, 17), and they become more differentiated toward the distal end (18) (Fig. 6*A*). First, we examined whether melanoblasts are present differentially in the eumelanin and pheomelanin region using immunohistochemistry (IHC) with an antibody to MITF (17), a marker for melanocyte progenitors. In both Partridge Plymouth Rock and Dark Cornish feathers, MITF^+^ melanoblasts are present in the collar before ramogenesis (barb branch formation) starts, and also in the early barb ridge, in both dark and light pigmented regions. In the late barb ridges, MITF becomes negative in well-differentiated melanocytes (Fig. 5) (MITF).

In situ hybridization (ISH) showed that *GJA5* was expressed in keratinocyte progenitors in the follicle collar. *GJA5* was also detected in melanoblasts just beneath the ramogenic zone (Fig. 5 *A* and *C*) (GJA5). In early barb ridges, *GJA5* was positive in both barbule epithelial cells and melanoblasts. This expression seems to be transient because the staining diminished in differentiated barb ridges toward the distal end. *ASIP* was undetectable using ISH in longitudinal sections. Cross-sections show weak *ASIP* expression in peripheral pulp adjacent to the rachis. The expression of *GJA5* and *ASIP* are similar in the pencilling feathers from Partridge Plymouth Rock hen (Fig. 5*A*) and double-lacing feathers from Dark Cornish hen (Fig. 5*C*).

Recent studies showed that while some longitudinal across-body pigmented stripe formation may be controlled by ASIP (19, 20), some longitudinal stripes can form through autonomous patterning role of melanocytes, forming melEM (melanoblast/cyte early marker) high and low stripes (19). Here we examined ISH of melEM and observed that they are expressed in the ramogenic zone, with high expression in eumelanin zone and low expression in pheomelanin zone. Thus, the intrafeather pigmented patterns of pencilling and double lacing here may also be patterned by two mechanisms: ASIP patterning in the rachis region and autonomous melanocyte patterning in the barb ridge region.

Fayoumi feathers show the autosomal barring pattern associated with lower *ASIP* expression in the peripheral pulp adjacent to the eumelanin region (3). For comparison, we carried out sections of Fayoumi feather follicle in the same experiment and they showed the typical patch of *ASIP* expression in the peripheral pulp adjacent to barb ridges (Fig. 5*B*).

In conclusion, we were not able to reveal a striking difference in *GJA5* expression between pencilling feathers from Partridge Plymouth Rock (*ml*^*+*^/*ml*^*+*^) and double-lacing feathers from Dark Cornish (*Ml/Ml*). However, it should be noted that we could not distinguish different isoforms of *GJA5* and that more subtle differences in expression levels could not be detected using the limited number of individuals used for IHC and ISH.

### Within-Feather Patterns in the Back-Cross Population Are Associated with the Presence of Mutant *MC1R* Alleles.

Recently we reported that autosomal barring as well as other periodic within-feather patterns, like double lacing and pencilling, are dependent on the presence of activating *MC1R* missense mutations (3). We therefore analyzed the *MC1R* alleles segregating in our intercross and found that the Partridge Plymouth Rock parents were homozygous for one of the previously described *Brown* alleles (1) that we designated *B1*, while three different *MC1R* alleles were present among the Dark Cornish parents, which corresponded to the previously reported *B2* (1), *BC* (1), and *UN* (21) alleles (*SI Appendix*, Table S7). These alleles carry multiple amino acid substitutions compared with the reference sequence (*SI Appendix*, Table S7). Most importantly, *Brown 1* and *2* carry the activating Glu92Lys mutation (22), while *BC2* and *UN2* carry the Leu133Pro mutation and probably also an activating mutation. The result is consistent with the assumption that the presence of activating *MC1R* mutations is required for variant forms of within-feather patterns in chickens. The four *MC1R* genotypes found in this intercross had an indistinguishable effect on pigmentation and segregation at this locus did not explain the deviation from full penetrance for the *Ml* allele (*SI Appendix*, Table S8). However, there was a tendency for a possible involvement of *MC1R* because all individuals classified as double recombinant or intermediate were genotyped as *B1/B1* or *B1/B2* (*SI Appendix*, Table S8) and, thus, homozygous for the Glu92Lys mutation (*P* = 0.03, Fisher’s exact test).

In conclusion, all four *MC1R* mutant alleles segregating in this cross may contribute to a melanotic phenotype like double lacing in the presence of *Ml*. The *B1* allele inherited from the Partridge Plymouth Rock parents was found both in the homozygous and heterozygous condition among double-lacing progeny and the three alleles (*B2*, *BC*, and *UN*) segregate in Dark Cornish chicken that uniformly show a double-lacing phenotype.

To test whether activating *MC1R* mutations are associated with Melanotic phenotypes, we investigated the *MC1R* alleles based on the WGS data of the 12 *Ml*/*−* samples used in IBD mapping (*SI Appendix*, Table S3). All 12 samples carry at least one copy of an activating *MC1R* allele (i.e., with Glu92Lys or Leu133GlnPro mutations). This supports the notion that activating *MC1R* mutations are necessary for the expression of variant periodic within-feather patterns, including Melanotic phenotypes.

## Discussion

This study provides evidence that *Melanotic* is caused by a *cis*-acting regulatory mutations affecting the expression of *GJA5* transcripts. The conclusion is based on classic linkage mapping that assigned *Melanotic* to an 820-kb region on chromosome 1, followed by high-resolution IBD mapping to a 21.4-kb region overlapping *GJA5* around position 93.85 Mb (Fig. 3*A*). The region harbors only one sequence variant, InDel1, that was unique to the *Melanotic* haplotype and not found on any wild-type haplotype. There was complete concordance between the presence of this mutation and a *Melanotic*-associated phenotype across 59 Melanotic and 136 nonmelanotic samples representing 25 breeds of chicken (Table 1). In InDel1, a 41-bp sequence with weak sequence conservation among vertebrates is replaced by a 38-bp sequence of unknown origin. InDel1 overlaps an ATAC-seq peak (11) and this 41-bp/38-bp replacement may affect gene regulation. Previous linkage analyses mapped *Ml* to 10 cM from *Dark Brown* (*Db*) (9) and 46 cM from *Pea-comb* (*P*) (23) on chromosome 1. We now know that *Ml* corresponds to *GJA5* (94 Mb), *Db* corresponds to *SOX10* (51 Mb) (7), and *P* corresponds to *SOX5* (66 Mb) (24), so *Ml* is closer to *P* than to *Db*. The previous linkage analysis indicating a map distance of only 10 cM between *Db* to *Ml* was based on a small (*n* = 31) back-cross pedigree (9) and is thus uncertain. We documented incomplete dominance of *Ml* because some *Ml/ml*^*+*^ heterozygotes showed an intermediate phenotype consistent with a previous report (8), but also noted incomplete penetrance as some *Ml/ml*^*+*^ heterozygotes were classified as nonmelanotic.

The *Ml* mutation in chickens has not been reported to be associated with any pleiotropic effects besides its effect on pigmentation, and *Ml/Ml* homozygotes are fully viable. In contrast, deleterious mutations in human *GJA5* are associated with atrial fibrillation and mutant *Gja5* mice show atrial arrhythmias and altered conduction velocity (25). The most likely reason for this difference is that the chicken *GJA5* mutation only affects regulation of gene expression, maybe only in the feather follicle, whereas the human and mouse mutations impair protein function.

Real-time PCR analysis demonstrated that *Melanotic* affects the expression of at least two of four *GJA5* transcripts (Fig. 4). The most conclusive evidence comes from the allelic imbalance analysis using *Ml/ml*^*+*^ heterozygotes, revealing that while the *Ml* allele expresses the NM_205504.2 transcript at a lower level (80%) than *ml*^*+*^, it is associated with a fivefold up-regulation of the XM_015295951.2 transcript. Our raw qRT-PCR data show that the NM_205504.2 transcript is about 60-fold more abundant than the XM_015295951.2 transcript. Thus, the 20% reduction of the former transcript may be functionally more important than the fivefold up-regulation of the latter. However, it is very likely that the different *GJA5* isoforms, characterized by the use of different exon 1 sequences (Fig. 3*B*) show different expression patterns among cell types and perhaps during different stages of feather follicle development. Therefore, a full understanding of how *Melanotic* affects *GJA5* transcription during feather development cannot be achieved until this process has been studied in detail by ISH for different isoforms of *GJA5* mRNA or single-cell transcriptomics.

The importance of gap-junction proteins for pigmentation patterns in zebrafish are well established by natural and artificial mutants of *connexin 41.8*, an ortholog of *connexin 40/GJA5* in birds and mammals, affecting the establishment of pigment stripes in zebrafish (26–28). Although there are no previously documented mutations in gap-junction protein genes affecting pigmentation in birds or mammals, recently Inaba et al. (19) demonstrated that it is possible to alter black stripes on the back of quail embryos by manipulating melanocyte-specific *GJA5/connexin 40* expression. Overexpression of *GJA5/connexin 40* expanded yellow regions, producing pheomelanin, while overexpression of a dominant negative form increased the size of black regions, producing eumelanin on the back feathers of chicks. Our study shows that *connexin 40/GJA5* also contributes to the genetic basis for within-feather pigmentation patterns in chicken. As an eumelanization factor in chickens, *Melanotic* expands the black stripes in the developing feathers (8) (Fig. 1) and a possible molecular mechanism may be through down-regulation of *GJA5* expression in melanocytes (19). This was the case for the predominant transcript NM_205504.2, while the less-abundant transcript XM_015295951.2 was up-regulated. The gap-junction proteins constitute a large family of proteins and the different functions of homomeric and heteromeric connexons (formed by six connexins of the same or different protein units, respectively) are poorly understood (29), it is unknown how altered *GJA5* expression caused by *Melanotic* affects the communication abilities of gap junctions. The expression patterns of different *GJA5* isoforms in different cell types during feather development need to be characterized in detail before we fully understand how *GJA5* polymorphism affects feather patterning. In a coculture system using human cells, communication between keratinocytes and melanocytes through gap junctions maintains pigment production in melanocytes (30). Similar experiments should be carried out in chickens to explore how gap-junction function affects the formation of plumage patterns.

Although further investigations are needed, the present work has established a relationship between the altered expression of gap-junction protein GJA5/connexin 40 and within-feather pigmentation patterns in birds, which can be connected to previous studies on pattern formations in birds. The presence of certain *Extension/MC1R* alleles [*R(Fayoumi)*, *B*, *BC*, or *Wh*] altering MC1R signaling transforms a stippling feather pattern to the pencilling pattern involving clear eumelanic bands (1), as illustrated in Fig. 6*B*. The addition of *Melanotic* stabilizes the eumelanic bands and expands the spacing between them, thus transforms pencilling to double lacing in which the eumelanic bands are wider (Fig. 6*B*). By investigating *MC1R* alleles in the 104 back-cross individuals (*SI Appendix*, Table S8) and 12 Melanotic samples with WGS data (*SI Appendix*, Table S3), we confirmed that the expression of Melanotic phenotypes including pencilling and double lacing is associated with the presence of activating *MC1R* missense mutations (i.e., Glu92Lys and Leu133GlnPro). This result supports our recent conclusion that *MC1R* is a major locus affecting all periodic feather patterns in chickens, except sex-linked barring (3). *MC1R* alleles carrying missense mutations enhancing MC1R signaling are required for the expression of pencilling, autosomal barring, and single and double lacing (3), whereas differences between those periodic patterns can be partially explained by altered *GJA5* expression.

Periodic pigment patterns in birds can form at across-body scale or within-feather scale (31). Lin et al. (17) presented two different modes for within-feather pattern formation in chickens. In the first mode, unpigmented regions form due to absence of melanocytes. For example, in the developing feather of sex-linked barring, the melanocytes, labeled by MITF, is absent in the unpigmented region, which is caused by the cyclic presence and absence of melanocyte progenitors in the lower bulge region. The second mode, controlled by modulation of melanogenesis, can be seen in single-lacing and autosomal barring feathers. MITF^+^ melanocytes are present in both white or yellow and black barb ridges, suggesting that the patterning is produced by differential suppression of melanogenesis. In the peripheral pulp, expression of *ASIP* was found to be associated with the nonblack region in single feathers from adult Silver Laced Wyandotte chickens (17). In that study, ASIP-coated beads were successfully used to suppress pigmentation in feathers from these chickens. Furthermore, recently we demonstrated that the periodic pattern autosomal barring is caused by enhanced MC1R signaling and the pattern of *ASIP* expression affecting MC1R signaling (3).

The third mode is revealed by clues from a study on the formation of periodic longitudinal pigmented stripes on the embryonic skin of Japanese quail. In developing Japanese quail embryos, across-body pigmented stripes form on the dorsal trunk (Fig. 6 *C*, *Upper*). While somite transplantation studies showed that expression of ASIP in the dermis controls pigment stripe pattern (20), a careful comparison of the timing of ASIP stripe appearance and pigmented stripe formation showed these longitudinal pigment stripes appear in temporal waves and may form via different mechanisms. Dermal longitudinal ASIP stripes appear in at least three temporal waves, A1 to A3 (19). The dermal A1 ASIP wave forms before pigment stripes form. Yet dermal ASIP wave A2 and A3 form in coincidentally when the formation of periodic melEM^+^/melEM^−^ longitudinal stripes is detected. This result suggests the formation of melEM stripes is ASIP-independent. Then, what is the patterning mechanism? Inaba et al. (19) found GJA5/connexin 40 (connexin 40 is the protein coded by *GJA5*) is expressed in melanocyte progenitors in Japanese quails, in both the eumelanin and pheomelanin stripe regions. Using embryonic quail skin explant cultures, spacing between pigment stripes were shown to be reduced by gap-junction inhibitors. Furthermore, melanocyte-specific inhibition of GJA5 in ovo, driven by melanocytes expressing a dominant-negative form of connexin 40, also resulted in the reduction of spacing between pigment stripes. These functional experiments imply some pigment signaling inhibitors may be mediated by gap-junction communication (Fig. 6 *C*, *Lower*) (19). Thus, a network composed of melanocyte populations appears to have a GJA5-dependent, autonomous patterning role that instructs the periodic stripe pattern in developing quail (Fig. 6*D*). While dermal ASIP, a nonmelanocyte-autonomous mechanism, has also been reported to drive pheomelanin stripe formation in quail embryos (20), these two mechanisms may coexist through complex interactions between dermal cells and melanocytes that remain to be investigated.

Here, in our independent genetic analyses of within-feather pigment patterning, we found that *connexin 40/GJA5* is also involved in the within-feather pigment pattern formation (Fig. 6*B*). As shown in Fig. 6*B*, *MC1R* and *GJA5* mutations both strengthen the within-feather pattern. Therefore, the gap-junction–mediated signals could be directly involved in the *MC1R* pathway involving activators for melanogenesis, such as cAMP, IP3, or Ca^2+^, as hypothesized by Inaba et al. (19). More complicated scenarios are also possible; for example, inhibitors of melanogenesis other than ASIP and cells other than melanocytes may be involved. Since MITF expression showed that melanoblasts are present in the yellow region, they are capable of expressing pheomelanin later, either through the noncanonical MC1R pathway, such as melanosome pH control by soluble adenylyl cyclase (32), or a later induced ASIP, as seen in quail stripes (31).

Within-feather pigmentation patterns in chickens and other ground-nesting birds provide camouflage that is of critical importance for survival. Our previous genetic studies established that genetic polymorphism at the *MC1R* locus and the interaction between the MC1R receptor and its antagonist ASIP have a primary role in generating within-feather pigmentation patterns in chicken (3). This requires cell–cell interactions because MC1R and ASIP are expressed on different cell types, primarily melanocytes and fibroblasts, respectively. While the molecular mediators of such cell–cell interactions remain to be identified, the present study advances our understanding of the mechanisms controlling camouflage colors in birds because it demonstrates that the activity of *connexin 40/GJA5* can modulate the periodic pigmentation patterns within individual feathers.

## Methods

The methods are described in detail in *SI Appendix*, *Supplementary Text*, which includes sections covering the following: animals, SNP-MaP analysis, WGS, linkage mapping, diagnostic test, quantitative real-time RT-PCR, allelic imbalance test, section immunostaining and ISH, reporter assay, and TRANSFAC analysis. The animal procedures used in this study were approved by the Institutional Animal Care and Use Committee at Virginia Polytechnic Institute and State University.

## Supplementary Material

Supplementary File

## Data Availability

The nucleotide sequences have been deposited in the National Center for Biotechnology Information BioProject (https://www.ncbi.nlm.nih.gov/bioproject/PRJNA679793) (33).
