# Supplementary material for: Cis-acting mutation affecting GJA5 transcription is underlying the Melanotic within-feather pigmentation pattern in chickens
Source: Proc Natl Acad Sci U S A. 2021 Oct 4;118(41):e2109363118. doi: 10.1073/pnas.2109363118 (PMC8521658; doi:10.1073/pnas.2109363118)
Supplement: Supplementary File [file pnas.2109363118.sapp.pdf]

Supplementary Information Appendix

Supplementary Text: Methods

Supplementary Fig. S1

Supplementary Tables S1-8

Supplementary Text

Methods

Animals: The animal procedures used in this study were approved by the Institutional Animal Care and Use Committee at Virginia Polytechnic Institute and State University. A linkage mapping population was initiated by crossing two Partridge Plymouth Rock males with four Dark Cornish females, all purchased from Murray McMurray Hatchery (www.mcmurrayhatchery.com, Webster City, Iowa, USA). Backcross matings between 17 F_1 females and 2 F_0 Partridge Plymouth Rock males produced 126 female progenies. Only females were considered in the analysis of the backcross population because pencilling or double lacing patterns are not observed in males. At 12 weeks of age, chickens with pencilling (n = 60), double lacing (n = 43), or intermediate patterns (n = 23) in the backcross population were classified by the same individual. Tissue samples (feather follicles, hypothalamus, intestine, liver, lung, muscle, and skin) for RNA isolation, immunostaining, and *in situ* hybridization were collected from two adult Dark Cornish hens and two adult Partridge Plymouth Rock hens. Feather follicle samples for RNA isolation were also collected from our in-house chicken population, which are three F_1 individuals from the mating between red junglefowl and Silver Sebright. The tissue sample collections followed the guidelines of Institutional Animal Care and Use Committee at Texas A&M University College of Veterinary Medicine & Biomedical Sciences.

SNP-MaP analysis: Two DNA pools were constructed based on the phenotypes of the backcross individuals (Pool_pencilling and Pool_double_lacing). Each individual contributed 250 ng of genomic DNA to the pool. The *Ml* gene was initially mapped by estimating SNP allele frequencies using the two DNA pools. This was accomplished using a high-density 600K chicken SNP genotyping array (by Affymetrix, 560,086 SNPs in total) and data were analyzed using the SNP-MaP (SNP Microarray and Pooling) approach (1). The genotyping array provided an intensity reading for the two alleles (X and Y) at each of the 560,086 SNPs that were reliably scored in this material. Relative allele frequencies (RAF) at each SNP for each DNA pool were calculated as X/(X + Y), where X and Y represented the intensity signals of each allele. For each SNP, absolute RAF differences (absRAFdif) were calculated by pairwise comparisons between Pool_pencilling and Pool_double_lacing. The absRAFdif was then plotted against SNP genomic locations (2). The absRAFdif value of a SNP was expected to peak in the vicinity of *Ml*.

Whole genome sequencing (WGS): Two DNA pools were constructed using the parental individuals in our mapping population (Pool_Cornish and Pool_Plymouth_Rock). These were subjected to whole genome sequencing together with two pools of backcross individuals (Pool_pencilling and Pool_double_lacing). Each of the four pools were sequenced to 30X coverage on Illumina HiSeqX with 2x150 bp paired-end reads.

These WGS data, except the two pools of backcross individuals, together with publicly available WGS data from 92 individuals or pooled samples were analyzed (*SI Appendix*, Table S3). The latter included 11 samples presumably fixed for the *Ml* allele

whereas the remaining 81 samples carried the wild-type allele ml^{+}. All Illumina paired-end FASTQ data were aligned to the red junglefowl genome assembly version GalGal6 using BWA (version: 0.7.12) (3), sorted with SAMtools (version: 1.6) (4), and variants were called with GATK HaplotypeCaller 3.8 (5). Structural variants were called with Lumpy (version: 0.2.13) (6). Sequence data from the backcross population (Pool_pencilling and Pool_double_lacing) were analyzed according to the same procedure, and were used to calculate pairwise F_{ST} values with Popoolation2 (7) in a sliding window approach with window size of 50 kb and step size of 10 kb and pool sizes of 60 pencilling individuals: 43 double lacing individuals. The F_{ST} values were Z-transformed and plotted against genomic locations.

Linkage mapping: The absRAFdif from SNP-MaP are arbitrary, because of possible microarray-based intensity variation and potential imprecision when construction DNA pools (8). Therefore, individual genotyping was necessary to confirm the allele frequencies and further reduce the candidate region. For the first round of linkage mapping, we selected six SNPs (*SI Appendix*, Table S1) with relatively high absRAFdif values (when comparing the backcross populations, the maximum absRAFdif is 0.5 in theory) within the peak region on chromosome 1 for further analysis, via the Kompetitive allele-specific PCR assay (KASP), developed by LGC Genomics (Beverly, MA, USA; www.lgcgenomics.com) (9). The six SNPs were genotyped using individual samples of all the F_0, F_1, and backcross females with pencilling or double lacing phenotypes, from the linkage mapping population. KASP assays were conducted following the manual with minor modifications. Genotyping results were validated by at least two replicates for each sample. Linkage mapping was carried out using the CRIMAP software (10) including the calculation of map distances and log10 odds ratio (LOD) between individual SNPs and *Melanotic*.

Within the candidate region defined by two of these six SNPs, ten more SNPs, fixed for different alleles in the parental lines and identified by whole genome sequencing, were selected for genotyping (*SI Appendix*, Table S2). These ten SNPs were used for a second round of linkage mapping, using only five recombinant backcross individuals via standard PCR and Sanger sequencing.

Diagnostic test: After the analysis of the candidate region based on the second round of linkage mapping, six sequence variants were identified as candidate causal mutations, which includes two InDels and four SNPs (*SI Appendix*, Table S4). A collection of 101 DNA samples from 52 different populations of chickens was studied focusing only on these six mutations. Nineteen of these populations carry *Melanotic* while the remaining thirty-three are wild-type at the *Ml* locus (*SI Appendix*, Table S5). Each of these samples were genotyped by fragment analysis following standard PCR amplifications for the two InDels in *SI Appendix*, Table S4. Three PCR primers were used for genotyping each of the two InDels (*SI Appendix*, Table S6), following the standard protocol of Platinum™ Green Hot Start PCR Master Mix (2X) (Invitrogen) with minor modifications. With the similar PCR protocol, a pair of primers for amplifying and sequencing the *MC1R* coding region (*SI Appendix*, Table S6) were also used to genotype all backcross individuals in the mapping population. Each of the samples listed in *SI Appendix*, Table S5 were genotyped via KASP for the four SNPs in *SI Appendix*, Table S4.

Quantitative real-time RT-PCR: Total RNA was extracted using Quick-RNA Miniprep Plus Kit (Zymo Research). First-strand cDNA was synthesized using SuperScript™ IV VILO™ Master Mix (Invitrogen). qPCRs were conducted with PowerUp™ SYBR™ Green Master Mix (Applied Biosystems). The products were detected with Bio-Rad CFX384 Touch™ Real-Time PCR Detection System. Details of the qPCR analysis including primer sequences are listed in Table S6. Each qPCR assay was carried out using three technical replicates.

Allelic imbalance test: Total RNA was extracted using Quick-RNA Miniprep Plus Kit (Zymo Research). First-strand cDNA was synthesized using SuperScript™ IV VILO™ Master Mix (Invitrogen). Two PCR primers were designed to quantify the allelic imbalance of *GJA5* expression: genomic DNA (gDNA) was amplified using GJA5_AI_F and GJA5_AI_R, while cDNA was amplified using GJA5_A_F or GJA5_B_F (for two different transcripts of *GJA5*) with the same reverse primer, respectively (*SI Appendix*, Table S6). PCR products from three cDNA samples which came from the three F_1 individuals together with PCR products from gDNA of the same three individuals, were Sanger sequenced using the same reverse primer. The peak heights for three SNPs, rs313638830 and rs731128040 found in transcript NM_205504.2 and rs312762853 found in transcript XM_015295951.2, were quantified using the PeakPicker 2 software (11). Allelic imbalance was estimated as the ratios of the peak height of the *Ml* allele over the ml^+ allele in the cDNA or gDNA.

Section immunostaining and *in situ* hybridization: Fixed embryos were embedded in paraffin and sectioned at 6-7 µm. After de-paraffinization, sections were processed to immunohistochemistry or *in situ* hybridization. The antibody to MITF was from Abcam (ab122982). The peroxidase staining was used after primary antibody treatment with procedures described (12).

Non-radioactive *in situ* hybridization was performed according to procedures previously described (13). Briefly, the sections were treated with proteinase K (10 µg/ml in PBS) for 20 min, re-fixed with 0.2% glutaraldehyde/4% paraformaldehyde, and rinsed with PBT. The sections were then prehybridized in hybridization buffer (containing 50% formamide, 5x sodium citrate/sodium chloride buffer, 1% sodium dodecyl sulfate, 50 µg/ml heparin, 50 µg/ml tRNA) at 65°C for one hour. After prehybridization, sections were placed in new prehybridization buffer containing 1-3 µg/ml digoxigenin-labeled riboprobes and hybridized overnight at 65°C. Finally, sections were incubated with alkaline phosphatase conjugated anti-digoxigenin Fab (Roche, Indianapolis, IN) overnight. Positive signals were detected by incubating the specimens with NBT/BCIP substrates (Promega, Madison). *In situ* hybridization primers for chicken MelEM (*GSTA3*) are: GG_GSTA3_F (5'-GGGAAGGATCTGAAGGAGAG-3') and GG_GSTA3_R (5'-TGAAGAAAGACACATCACAA-3'). Primers for *ASIP* and *GJA5* are according to Inaba *et al.* (14).

Reporter assay: Previous Cap Analysis of Gene Expression (CAGE) analysis detected the position of one of the transcription start sites (TSSs) of chicken *GJA5* using mRNA from whole embryo samples (15). The 1,869 bp sequence (*SI Appendix*, Fig. S1A)

upstream from this TSS in wild-type and mutant *GJA5* alleles, including InDel1 and SNP rs316201461, were synthesized *in vitro* and cloned into the promoterless Firefly Luciferase vector pGL4.15[luc2P/Hygro] by Genscript (Leiden, Netherlands). pRL-TK plasmid monitoring the transfection efficiency was purchased from Promega (E2241). Five ng of each plasmid was used to transform the XL1-Blue competent cells (Agilent, 200236), and plasmid DNA was subsequently prepared from 200 ml overnight culture with an EndoFree plasmid Maxi Kit (Qiagen).

DF-1 chicken fibroblast cells (16) were a gift from Dr. Benjamin Schusser (Technical University of Munich). The DF-1 cells were maintained in DMEM supplemented with 10% FBS, 100 U/ml penicillin, 100 μg/ml streptomycin and 292 μg/ml L-Glutamine (ThermoFisher Scientific) at 39°C with 5% CO_2. On day 1, an aliquot of 2.5 X 10^4 cells/well were seeded in a 48-well plate. On the next day, a plasmid mixture containing 247.5 ng pGL4.15[luc2P/Hygro] with or without GJA5 promoters and 2.5 ng pRL-TK was applied to transfect each well of cells using jetPRIME transfection reagent (Polyplus). Four replicates were used for each promoter construct. Medium was replaced by fresh medium 4 h after transfection. On day 3, luminescence of each well was analyzed using a Dual-Luciferase Reporter assay (Promega) on an Infinite M200 Microplate Reader (Tecan Group Ltd., Switzerland), and the luciferase activity was represented as the ratio of firefly (pGL4.15[luc2P/Hygro]) to Renilla (pRL-TK) luminescence.

TRANSFAC analysis: Predictions of putative transcription factor binding sites were done by the MATCH program in the TRANSFAC database (17). The vertebrate database was used and only predicted binding sites with a matrix score of 1.0 were selected for further analysis.

Literature cited in Supplementary Text

1. S. J. Docherty, L. M. Butcher, L. C. Schalkwyk, R. Plomin, Applicability of DNA pools on 500 K SNP microarrays for cost-effective initial screens in genomewide association studies. *BMC Genomics* **8**, 214 (2007).
2. K. L. Wells, *et al.*, Genome-wide SNP scan of pooled DNA reveals nonsense mutation in *FGF20* in the scaleless line of featherless chickens. *BMC Genomics* **13**, 257 (2012).
3. H. Li, Aligning sequence reads, clone sequences and assembly contigs with BWA-MEM. *arXiv Prepr. arXiv1303.3997* (2013) (January 12, 2021).
4. H. Li, *et al.*, The Sequence Alignment/Map format and SAMtools. *Bioinformatics* **25**, 2078–2079 (2009).
5. R. Poplin, *et al.*, Scaling accurate genetic variant discovery to tens of thousands of samples. *bioRxiv. https://doi.org/10.1101/201178* (2017) https:/doi.org/10.1101/201178.

6. R. M. Layer, C. Chiang, A. R. Quinlan, I. M. Hall, LUMPY: A probabilistic framework for structural variant discovery. *Genome Biol.* **15**, R84 (2014).

7. R. Kofler, R. V. Pandey, C. Schlotterer, PoPoolation2: identifying differentiation between populations using sequencing of pooled DNA samples (Pool-Seq). *Bioinformatics* **27**, 3435–3436 (2011).

8. S. Macgregor, Most pooling variation in array-based DNA pooling is attributable to array error rather than pool construction error. *Eur. J. Hum. Genet.* **15**, 501–504 (2007).

9. K. Semagn, R. Babu, S. Hearne, M. Olsen, Single nucleotide polymorphism genotyping using Kompetitive Allele Specific PCR (KASP): overview of the technology and its application in crop improvement. *Mol. Breed.* **33**, 1–14 (2014).

10. P. Green, K. Falls, S. Crooks, *Documentation for CRIMAP, version 2.4* (Washington University School of Medicine, 1990) (January 10, 2020).

11. B. Ge, *et al.*, Survey of allelic expression using EST mining. *Genome Res.* **15**, 1584–1591 (2005).

12. S. J. Lin, *et al.*, Topology of feather melanocyte progenitor niche allows complex pigment patterns to emerge. *Science.* **340**, 1442–1445 (2013).

13. A. Li, *et al.*, Diverse feather shape evolution enabled by coupling anisotropic signalling modules with self-organizing branching programme. *Nat. Commun.* **8**, 1–13 (2017).

14. M. Inaba, *et al.*, Instructive role of melanocytes during pigment pattern formation of the avian skin. *Proc. Natl. Acad. Sci. U. S. A.* **116**, 6884–6890 (2019).

15. M. Lizio, *et al.*, Systematic analysis of transcription start sites in avian development. *PLOS Biol.* **15**, e2002887 (2017).

16. M. Himly, D. N. Foster, I. Bottoli, J. S. Iacovoni, P. K. Vogt, The DF-1 chicken fibroblast cell line: Transformation induced by diverse oncogenes and cell death resulting from infection by avian leukosis viruses. *Virology* **248**, 295–304 (1998).

17. A. E. Kel, *et al.*, MATCH^{TM}: a tool for searching transcription factor binding sites in DNA sequences. *Nucleic Acids Res.* **31**, 3576–3579 (2003).

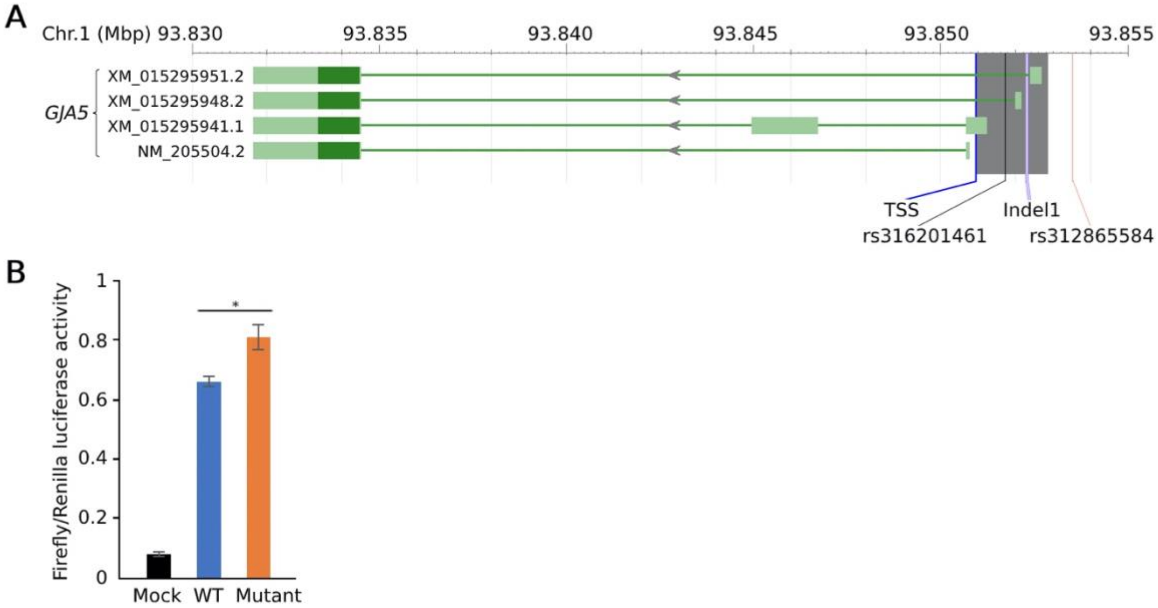

Fig. S1. Reporter assay suggesting a regulatory effect of the *Ml* haplotype. (**A**) The dark grey area indicates the 1,869 bp sequence at the 5'end of *GJA5* used in the reporter assay, including one SNP and InDel1. TSS: predicted transcription start site (S. Foissac, *et al.*, Multi-species annotation of transcriptome and chromatin structure in domesticated animals. *BMC Biol.* **17**, 108 (2019)). (**B**) Results of dual-luciferase reporter assay represented as mean ± SEM of quadruplicate samples.

Table S1. First round of linkage mapping of *Melanotic* using individual SNP analysis

dbSNP	absRAFdif[a]	Position on Chr. 1 (bp)	Genetic distance from *Melanotic* (cM)[b]	LOD score[b]
rs313722403	0.42	87,572,229	15.8	11.7
rs316117302	0.50	90,527,754	3.9	23.7
rs314066698	0.49	93,048,147	1.9	26.7
rs314825166	0.45	94,632,771	2.9	25.1
rs13908401	0.41	98,040,916	8.7	17.8
rs317883641	0.37	101,886,057	10.7	15.8

[a] Calculated by contrasting RAF values for pencilling and double lacing DNA pools
[b] Calculated using the CRIMAP software, a LOD score > 3.0 is considered genome-wide significant

Table S2. Genetic markers used for the second round of linkage mapping of *Melanotic*

dbSNP	Position on Chr. 1 (bp)	Allele in Plymouth Rock	Allele in Cornish	Primer sequence (5’-3’)
rs314254723[a]	93,362,467	C	T	F: TCTCCAGCCTCCCTTATCCA<br>R: CCCGCAGATAGACACAACCA
rs316556553[a]	93,438,578	C	T	F: CTGGCTTTCCTGTTCCCTCTTA<br>R: CTCATCTCACTGCTCACCACCT
rs317325096[a]	93,577,739	T	C	F: ACTGCCGTTGCATTGTGTTC<br>R: AACCTTCAGGATAATGGCTGGA
rs15347589[a]	93,641,597	G	A	F: GAGAGGAGGCAGCGTGTCTT<br>R: CCCGACTCAACTTCCCTGTC
rs14859493	93,720,150	T	C	F: AACAAGGATGGTGGGCAATC<br>R: ATCCCCTCGCACCTACAGAG
rs316851065	93,757,350	T	G	F: CAGCTCCCTTCCTTCTGTTCAT<br>R: CCAGGGAGACGAGGAAACTAGA
rs13686931	93,811,196	A	G	F: ACACTGTGCCACCCTGTGTC<br>R: TGCAGATGTGACCCTCGAAC
rs13905107	93,918,761	C	T	F: GCAAATATCCCTCCCTGCAC<br>R: CATTGGAGGACTTGCTGCTG
rs317124570	94,255,352	A	G	F: CCAGCCCCAAACTCTTCTTG<br>R: AGCCATGCTCAGCTGTTTGA
rs317781986[a]	94,458,113	A	G	F: ACCAAACTCCAGCATCAGCA<br>R: TCCCATTACTGAAACGTGTGGA

[a] SNPs that show recombination in any of the five individuals used in the second round of linkage mapping. No recombination was found for the other SNPs, defining the candidate region of *Melanotic*.

Table S3. Public whole genome sequence data used in this study. This includes only samples that shows a periodic feather pattern because we cannot deduce the presence/absence of *Ml* in birds that show a non-periodic pattern like Buff, Columbian, and Black. The exception to this rule is Polish White Crested Black chicken because a previous study demonstrated that they carry *Ml* (J. W. Moore, J. R. Smyth, Melanotic: Key to a phenotypic enigma in the fowl. *J. Hered.* **62**, 215–219 (1971)). Information on *GJA5, Dark brown/SOX10* and *MC1R* were deduced from whole genome sequence data.

Breed[a]	Phenotype	Library type	BioSample accession	Genotype[b]	*GJA5* Indel1	*Dark brown SOX10*	*MC1R* mutation[c]
Dark Cornish	Double Laced	Pool	SAMN16846413	*Ml*/-	*Indel1*/*Indel1*	db^+/db^+	E92K or L133P
Dark Cornish	Double Laced	Individual	SAMEA6529968	*Ml*/-	*Indel1*/*Indel1*	db^+/db^+	L133P
Dark Cornish	Double Laced	Individual	SAMN13380102	*Ml*/-	*Indel1*/*Indel1*	db^+/db^+	E92K
Hamburg	Silver Spangled	Pool	SAMN16846357	*Ml*/-	*Indel1*/*Indel1*	db^+/db^+	E92K
Hamburg	Silver Spangled	Individual	SAMEA6529977	*Ml*/-	*Indel1*/*Indel1*	*Db*/*Db*	E92K
Polish	White Crested Black	Individual	SAMEA6529996	*Ml*/-	*Indel1*/*Indel1*	db^+/db^+	E92K
Polish	White Crested Black	Individual	SAMN03177328	*Ml*/-	*Indel1*/*Indel1*	db^+/db^+	E92K/L133Q
Sebright	Gold Laced	Individual	SAMEA6529948	*Ml*/-	*Indel1*/*Indel1*	*Db*/*Db*	E92K
Sebright	Silver Laced	Pool	SAMEA104432213	*Ml*/-	*Indel1*/*Indel1*	*Db*/*Db*	E92K
Sebright	Silver Laced	Pool	SAMN16846355	*Ml*/-	*Indel1*/*Indel1*	*Db*/*Db*	E92K
Sebright	Silver Laced	Individual	SAMEA6529949	*Ml*/-	*Indel1*/*Indel1*	*Db*/*Db*	E92K
Wyandotte	Silver Laced	Individual	SAMEA6529967	*Ml*/-	*Indel1*/*Indel1*	db^+/db^+	E92K
Buttercup	Autosomal barring	Pool	SAMN16846359	ml^+/ml^+	*WT*/*WT*	*Db*/*Db*	
Buttercup	Autosomal barring	Individual	SAMN03177329	ml^+/ml^+	*WT*/*WT*	db^+/db^+	
Cochin	Pencilling	Individual	SAMN12236899	ml^+/ml^+	*WT*/*WT*	db^+/db^+	
Cochin	Pencilling	Individual	SAMN12236900	ml^+/ml^+	*WT*/*WT*	db^+/db^+	
Cochin	Pencilling	Individual	SAMN12236901	ml^+/ml^+	*WT*/*WT*	db^+/db^+	
Cochin	Pencilling	Individual	SAMN12236902	ml^+/ml^+	*WT*/*WT*	db^+/db^+	
Cyan-shank Partridge	Pencilling	Pool	SAMN12990250	ml^+/ml^+	*WT*/*WT*	db^+/db^+	
East Friesian Gull	Pencilling	Pool	SAMEA104432208	ml^+/ml^+	*WT*/*WT*	*Db*/*Db*	
Fayoumi	Autosomal barring	Pool	SAMN06612097	ml^+/ml^+	*WT*/*WT*	*Db*/*Db*	
Fayoumi	Autosomal barring	Pool	SAMN16846360	ml^+/ml^+	*WT*/*WT*	*Db*/*Db*	
Indochinese	Stippling	Pool	SAMEA104432194	ml^+/ml^+	*WT*/*WT*	db^+/db^+	

RJF						
Java RJF	Stippling	Individual	SAMD00077852	ml^+/ml^+	*WT*/*WT*	db^+/db^+
Java RJF	Stippling	Individual	SAMD00077853	ml^+/ml^+	*WT*/*WT*	db^+/db^+
Java RJF	Stippling	Individual	SAMD00077854	ml^+/ml^+	*WT*/*WT*	db^+/db^+
Plymouth Rock	Pencilling	Pool	SAMN16846358	ml^+/ml^+	*WT*/*WT*	db^+/db^+
RJF	Stippling	Individual	SAMN02333832	ml^+/ml^+	*WT*/*WT*	db^+/db^+
RJF	Stippling	Individual	SAMN02333833	ml^+/ml^+	*WT*/*WT*	db^+/db^+
RJF	Stippling	Individual	SAMN02486161	ml^+/ml^+	*WT*/*WT*	db^+/db^+
RJF	Stippling	Individual	SAMN02712039	ml^+/ml^+	*WT*/*WT*	db^+/db^+
RJF	Stippling	Individual	SAMN02712040	ml^+/ml^+	*WT*/*WT*	db^+/db^+
RJF	Stippling	Individual	SAMN02712041	ml^+/ml^+	*WT*/*WT*	db^+/db^+
RJF	Stippling	Individual	SAMN02712042	ml^+/ml^+	*WT*/*WT*	db^+/db^+
RJF	Stippling	Individual	SAMN02712045	ml^+/ml^+	*WT*/*WT*	db^+/db^+
RJF	Stippling	Individual	SAMN10471716	ml^+/ml^+	*WT*/*WT*	db^+/db^+
RJF	Stippling	Individual	SAMN14651083	ml^+/ml^+	*WT*/*WT*	db^+/db^+
RJF	Stippling	Individual	SAMN14814745	ml^+/ml^+	*WT*/*WT*	db^+/db^+
RJF	Stippling	Individual	SAMN14814771	ml^+/ml^+	*WT*/*WT*	db^+/db^+
RJF	Stippling	Individual	SAMN14814772	ml^+/ml^+	*WT*/*WT*	db^+/db^+
RJF	Stippling	Individual	SAMN14814774	ml^+/ml^+	*WT*/*WT*	db^+/db^+
RJF	Stippling	Individual	SAMN14814775	ml^+/ml^+	*WT*/*WT*	db^+/db^+
RJF	Stippling	Individual	SAMN14814776	ml^+/ml^+	*WT*/*WT*	db^+/db^+
RJF	Stippling	Individual	SAMN14814777	ml^+/ml^+	*WT*/*WT*	db^+/db^+
RJF	Stippling	Individual	SAMN14814778	ml^+/ml^+	*WT*/*WT*	db^+/db^+
RJF	Stippling	Individual	SAMN14814779	ml^+/ml^+	*WT*/*WT*	db^+/db^+
RJF	Stippling	Individual	SAMN14814781	ml^+/ml^+	*WT*/*WT*	db^+/db^+
RJF	Stippling	Individual	SAMN14814782	ml^+/ml^+	*WT*/*WT*	db^+/db^+
RJF	Stippling	Individual	SAMN14814784	ml^+/ml^+	*WT*/*WT*	db^+/db^+
RJF	Stippling	Individual	SAMN14814785	ml^+/ml^+	*WT*/*WT*	db^+/db^+
RJF	Stippling	Individual	SAMN14814786	ml^+/ml^+	*WT*/*WT*	db^+/db^+
RJF	Stippling	Individual	SAMN14814788	ml^+/ml^+	*WT*/*WT*	db^+/db^+
RJF	Stippling	Individual	SAMN14814789	ml^+/ml^+	*WT*/*WT*	db^+/db^+
RJF	Stippling	Individual	SAMN14814790	ml^+/ml^+	*WT*/*WT*	db^+/db^+
RJF	Stippling	Individual	SAMN14814791	ml^+/ml^+	*WT*/*WT*	db^+/db^+
RJF	Stippling	Individual	SAMN14814792	ml^+/ml^+	*WT*/*WT*	db^+/db^+
RJF	Stippling	Individual	SAMN14814793	ml^+/ml^+	*WT*/*WT*	db^+/db^+
RJF	Stippling	Individual	SAMN14814795	ml^+/ml^+	*WT*/*WT*	db^+/db^+
RJF	Stippling	Individual	SAMN14814796	ml^+/ml^+	*WT*/*WT*	db^+/db^+
RJF	Stippling	Individual	SAMN14814797	ml^+/ml^+	*WT*/*WT*	db^+/db^+
RJF	Stippling	Individual	SAMN14814798	ml^+/ml^+	*WT*/*WT*	db^+/db^+
RJF	Stippling	Individual	SAMN14814799	ml^+/ml^+	*WT*/*WT*	db^+/db^+
RJF	Stippling	Individual	SAMN14814802	ml^+/ml^+	*WT*/*WT*	db^+/db^+
RJF	Stippling	Individual	SAMN14814804	ml^+/ml^+	*WT*/*WT*	db^+/db^+
RJF	Stippling	Individual	SAMN14814805	ml^+/ml^+	*WT*/*WT*	db^+/db^+
RJF	Stippling	Individual	SAMN14814806	ml^+/ml^+	*WT*/*WT*	db^+/db^+

RJF	Stippling	Individual	SAMN14814808	ml^+/ml^+	*WT/WT*	db^+/db^+
RJF	Stippling	Individual	SAMN14814809	ml^+/ml^+	*WT/WT*	db^+/db^+
Sumatra RJF	Stippling	Individual	SAMD00077855	ml^+/ml^+	*WT/WT*	db^+/db^+
Sumatra RJF	Stippling	Individual	SAMD00077856	ml^+/ml^+	*WT/WT*	db^+/db^+
Thailand RJF	Stippling	Individual	SAMEA5160200	ml^+/ml^+	*WT/WT*	db^+/db^+
Thailand RJF	Stippling	Individual	SAMEA5160201	ml^+/ml^+	*WT/WT*	db^+/db^+
Thailand RJF	Stippling	Individual	SAMEA5160202	ml^+/ml^+	*WT/WT*	db^+/db^+
Thailand RJF	Stippling	Individual	SAMEA5160203	ml^+/ml^+	*WT/WT*	db^+/db^+
Thailand RJF	Stippling	Individual	SAMEA5160204	ml^+/ml^+	*WT/WT*	db^+/db^+
Thailand RJF	Stippling	Individual	SAMEA5160205	ml^+/ml^+	*WT/WT*	db^+/db^+
Thailand RJF	Stippling	Individual	SAMEA5160206	ml^+/ml^+	*WT/WT*	db^+/db^+
Thailand RJF	Stippling	Individual	SAMEA5160207	ml^+/ml^+	*WT/WT*	db^+/db^+
Thailand RJF	Stippling	Individual	SAMEA5160208	ml^+/ml^+	*WT/WT*	db^+/db^+
Thailand RJF	Stippling	Individual	SAMEA5160209	ml^+/ml^+	*WT/WT*	db^+/db^+
Thailand RJF	Stippling	Individual	SAMEA5160210	ml^+/ml^+	*WT/WT*	db^+/db^+
Thailand RJF	Stippling	Individual	SAMEA5160211	ml^+/ml^+	*WT/WT*	db^+/db^+
Thailand RJF	Stippling	Individual	SAMEA5160212	ml^+/ml^+	*WT/WT*	db^+/db^+
Thailand RJF	Stippling	Individual	SAMEA5160213	ml^+/ml^+	*WT/WT*	db^+/db^+
Thailand RJF	Stippling	Individual	SAMEA5160214	ml^+/ml^+	*WT/WT*	db^+/db^+
Thailand RJF	Stippling	Individual	SAMEA5160215	ml^+/ml^+	*WT/WT*	db^+/db^+
Thailand RJF	Stippling	Individual	SAMEA5160216	ml^+/ml^+	*WT/WT*	db^+/db^+
Thailand RJF	Stippling	Individual	SAMEA5160217	ml^+/ml^+	*WT/WT*	db^+/db^+
Thailand RJF	Stippling	Individual	SAMEA5160218	ml^+/ml^+	*WT/WT*	db^+/db^+
Thailand RJF	Stippling	Individual	SAMEA5160219	ml^+/ml^+	*WT/WT*	db^+/db^+
Thailand RJF	Stippling	Individual	SAMEA5160220	ml^+/ml^+	*WT/WT*	db^+/db^+
Thailand RJF	Stippling	Individual	SAMEA5160221	ml^+/ml^+	*WT/WT*	db^+/db^+
Thailand RJF	Stippling	Individual	SAMEA5160222	ml^+/ml^+	*WT/WT*	db^+/db^+
Thailand RJF	Stippling	Individual	SAMEA5160223	ml^+/ml^+	*WT/WT*	db^+/db^+

[a] RJF = red junglefowl
[b] The *Ml/-* and ml^+/ml^+ genotype was inferred from plumage phenotype.
[c] Only the presence of the activating mutations E92K and L133Q/P are presented for *Ml/-* individuals; a careful analysis of *MC1R* variation among all the ml^+/ml^+ birds were outside the scope of the present study.

Table S4. Sequence variants strongly associated with *Melanotic* and located within the IBD region on chicken chromosome 1

Position on Chr.1 (bp)	Allele in Plymouth Rock	Allele in Cornish	dbSNP or sequence
93,851,717	C	A	rs316201461
93,852,277-93,852,317	InDel1-WT	InDel1-*Ml*	InDel1-WT: TGAGGGTCAAAGAGAGTGCA-TGATCTCTGGAAAGGCCTAGG<br>InDel1-*Ml*: AAACTACTGCTTATTAAAC-TACTACTACTATTTAAGAT
93,853,513	T	A	rs312865584
93,857,288-93,857,293	InDel2-WT	InDel2-*Ml*	InDel2-WT: GTCTGC<br>InDel2-*Ml*: TCCTTGGTCCT
93,865,772	C	T	rs317670985
93,866,099	C	T	rs794497277

Table S5. List of DNA samples used for diagnostic tests for six candidate mutations of *Ml*

Breed	Variety	Source	Number of animals	Genotype[a]
American Longtail	Wild-type	H & H Longtails	2	ml^+/ml^+
B (Wild-type)	Wild-type	Tucson, AZ, U.S.	2	ml^+/ml^+
Brahma	Dark	Ideal Poultry	1	ml^+/ml^+
Brahma	Dark	Lucasville, OH, U.S.	1	ml^+/ml^+
Buttercup	Buttercup	Lucasville, OH, U.S.	2	ml^+/ml^+
Buttercup	Buttercup	Murray McMurray	2	ml^+/ml^+
Campine	Golden	Raleigh, NC, U.S.	1	ml^+/ml^+
Campine	Silver Pencilled	Canterbury, U.K.	2	ml^+/ml^+
Chantecler	Partridge	Lucasville, OH, U.S.	1	ml^+/ml^+
Cochin	Golden laced	Lucasville, OH, U.S.	1	*Ml*/-
Cochin	Partridge	Murray McMurray	2	ml^+/ml^+
Cornish	Dark	Murray McMurray	6	*Ml*/-
Czech	Golden	Canterbury, U.K.	2	ml^+/ml^+
Fayoumi	Barred	East Lansing, MI, U.S.	1	ml^+/ml^+
Fayoumi	Barred	Ideal Poultry	2	ml^+/ml^+
Fayoumi	Barred	Ames, IA, U.S.	2	ml^+/ml^+
Fayoumi	Barred	Ames, IA, U.S.	2	ml^+/ml^+
Hamburg	Golden Pencilled	Murray McMurray	2	ml^+/ml^+
Hamburg	Silver Spangled	Cackle Hatchery	1	*Ml*/-
Hamburg	Silver Spangled	Macclesfield, NC, U.S.	2	*Ml*/-
Hamburg	Silver Spangled	Murray McMurray	2	*Ml*/-
Hamburg	Silver Spangled	Canterbury, U.K.	2	*Ml*/-
Red junglefowl	Red	Richardson line	2	ml^+/ml^+
Red junglefowl	Red	Raleigh, NC, U.S.	2	ml^+/ml^+
Malay	Red	Ideal Poultry	1	ml^+/ml^+
New Hampshire	Red	Ideal Poultry	1	ml^+/ml^+
New Hampshire	Red	Canterbury, U.K.	2	ml^+/ml^+
Phoenix	Golden	Ideal Poultry	1	ml^+/ml^+
Phoenix	Silver	Marshfield, WI, U.S.	1	ml^+/ml^+
Phoenix	Silver Duckwing	Ideal Poultry	1	ml^+/ml^+
Plymouth Rock	Partridge	Murray McMurray	2	ml^+/ml^+
Plymouth Rock	Silver pencilled	Murray McMurray	2	ml^+/ml^+
Polish[b]	Buff laced	Macclesfield, NC, U.S.	2	*Ml*/-
Polish[b]	White Crested Black	Murray McMurray	1	*Ml*/-
Polish[b]	White Crested Black	Canterbury, U.K.	1	*Ml*/-
Polish[b]	White Crested Black	Lucasville, OH, U.S.	1	*Ml*/-
Polish[b]	White Crested Black	Marshfield, WI, U.S.	1	*Ml*/-

Polish[b]	White Crested Black	Raleigh, NC, U.S.	2	*Ml*/-
Polish[b]	White Crested Blue	Lucasville, OH, U.S.	1	*Ml*/-
Rhode Island	Red	East Lansing, MI, U.S.	1	ml^{+}/ml^{+}
Rhode Island	Red	Macclesfield, NC, U.S.	2	ml^{+}/ml^{+}
Rhode Island	Red	Canterbury, U.K.	2	ml^{+}/ml^{+}
Saipan junglefowl	Wild-type	East Lansing, MI, U.S.	1	ml^{+}/ml^{+}
Sebright	Golden	Lucasville, OH, U.S.	1	*Ml*/-
Sebright	Golden	Macclesfield, NC, U.S.	2	*Ml*/-
Sebright	Silver	Macclesfield, NC, U.S.	2	*Ml*/-
Sebright	Silver	Murray McMurray	16	*Ml*/-
Sicilian Buttercup	Buttercup	Ideal Poultry	2	ml^{+}/ml^{+}
Spitzhauben	Spangled	Cackle Hatchery	1	*Ml*/-
Spitzhauben	Spangled	Raleigh, NC, U.S.	2	*Ml*/-
Wyandotte	Partridge	Murray McMurray	2	ml^{+}/ml^{+}
Wyandotte	Silver penciled	Murray McMurray	2	ml^{+}/ml^{+}

[a] Inferred from plumage phenotype

[b] As previously reported (J. W. Moore, J. R. Smyth, Melanotic: Key to a phenotypic enigma in the fowl. *J. Hered.* **62**, 215–219 (1971)) Polish chickens are *Ml*/*Ml*

Table S6. Primers used for diagnostic tests, qPCR, and allelic imbalance tests in this study

Primer name	Amplification target	Primer sequence (5'-3')	Product size (bp)	Tm (°C)
InDel1_F1	InDel1-WT	GAGGGTCAAAGAGAGTGCATGA	108	61
InDel1_R		GTATAAATTGAATGCAAATGCAAGG		
InDel1_F2	InDel1-mutant	CAGGTTAGATGCTTATTAAACTACT-GCTTA	123	
InDel2_R1	InDel2-WT	AGGACCAAGGCGCAGACTC	96	62
InDel2_F		AGCATCATTGGCCTGGAGA		
InDel2_R2	InDel2-mutant	CAACAGGACCAAGGCAGGAC	105	
GJA5_A_F	NM_205504.2	CCGCTCCTCCCTCCACCTGT	100	65
GJA5_A_R		TCCCCCATTTCTGCTCACCA		
MC1R_F	*MC1R*	CTTTGTAGGTGCTGCAGTTGTG	1,049	62
MC1R_R		ATCCATCCATCCTCCTGTCTGT		
GJA5_B_F	XM_015295951.2	GCTGTAAGAATACCTTACAAGCAA	135	60
GJA5_R[a]		TGTGGAGTGCTTGTGGACCTCCT		
GJA5_C_F	XM_015295948.2	TCAGTGCCTCTCAGCGTGTGAC	168	65
GJA5_D_F	XM_015295941.1	GGAGGCAGAAGAAAAGACACTGGA	140	65
GJA8_F1	XM_015295800.2 &	CCCAATGGCTTGTGTAAAACTTGGA	141	65
GJA8_R1[b]	XM_015295822.2	CCCAGGATCAGGATGCGGAAA		
GJA8_F2	All *GJA8* transcripts	GGTGACTGGAGTTTCTTGGGGAACA	110	65
GJA8_F3	All *GJA8* transcripts	TCGTGGTGGCTGCTGTGTCC	118	65
GJA8_R3		TCTGGCACCTCCCCCATCTG		
GJA8_F4	All *GJA8* transcripts	TTGAGCCCAGTCCCCTTCCA	127	65
GJA8_F4		TCCGGTTCCTTCGCTTGTGC		
Actin_F	NM_205518.1	TGCGTGACATCAAGGAGAAG	111	60 or 65
Actin_R		GACCATCAGGGAGTTCATAGC		
GJA5_AI_F	For allelic imbalance test	TGGTGGGGAAGGTCTGGTTGA	1035	65
GJA5_AI_R		CTGACCTCGCCTTGCTGCTG		

[a] During qPCR, GJA5_R was also used as the reverse primer with GJA5_C_F and GJA5_D_F, respectively.
[b] During qPCR, GJA8_R1 was also used as the reverse primer with GJA8_F2.

Table S7. Variable amino acid residues among *MC1R* alleles identified in pools of Partridge Plymouth Rock and Dark Cornish and confirmed by Sanger sequencing

Breed	Frequency[a]	Variant sites							Allele[b]
		71	92	133	137	143	213	215	
Reference	n.a.	Met	Glu	Leu	Ala	Thr	Cys	His	*WT*
Partridge Plymouth Rock	1	Thr	Lys	-	-	-	-	Pro	*B1*
Dark Cornish	0.62	-	Lys	-	Thr	-	-	-	*B2*
	0.25	-	-	Pro	-	Ala	Arg	-	*BC*
	0.13	-	-	Pro	-	-	Arg	-	*UN*

[a] Calculated based on the number of reads in pooled WGS data. n.a.=not applicable.
[b] *B1 (Brown1)*, *B2 (Brown2)*, and *BC* (*buttercup*) were all included in the summary by Andersson et al. ("The genetic basis for pigmentation phenotypes in poultry" in *Advances in Poultry Genetics and Genomics*, S. E. Aggrey, H. Zhou, M. Tixier-Boichard, D. D. Rhoads, Eds. (Burleigh Dodds Science Publishing, 2020), pp. 67–106.); *UN* (*uncharacterized*) has been reported by Guo *et al*. (Genetic variation of chicken *MC1R* gene in different plumage colour populations. *Br. Poult. Sci.* **51**, 734–739 (2010)).

Table S8. The distribution of *Ml* and *MC1R* genotypes among different phenotypes in the backcross population

<table>
<tr><th rowspan="2">Phenotype</th><th rowspan="2">Ml genotype[a]</th><th colspan="4">MC1R genotype</th><th rowspan="2">n</th><th rowspan="2" colspan="2">χ^2 test P-value</th></tr>
<tr><th>B1/B1</th><th>B1/B2</th><th>B1/BC</th><th>B1/UN</th></tr>
<tr><td>Double recombinant[b]</td><td>Ml/ml^{+}</td><td>5</td><td>4</td><td>0</td><td>0</td><td>9</td><td rowspan="2">0.48</td><td></td></tr>
<tr><td>Double lacing</td><td>Ml/ml^{+}</td><td>14</td><td>10</td><td>5</td><td>2</td><td>31</td><td rowspan="2">0.43</td></tr>
<tr><td rowspan="2">Intermediate</td><td>Ml/ml^{+}</td><td>6</td><td>4</td><td>0</td><td>0</td><td>10</td><td></td></tr>
<tr><td>ml^{+}/ml^{+}</td><td>8</td><td>1</td><td>0</td><td>0</td><td>9</td><td rowspan="2" colspan="2">0.22</td></tr>
<tr><td>Pencilling</td><td>ml^{+}/ml^{+}</td><td>23</td><td>17</td><td>4</td><td>1</td><td>45</td></tr>
</table>

[a] Based on genotyping InDel1.
[b] The backcross individuals that show pencilling pattern but carry the *Ml* haplotype.
